# Can the buck always be passed to the highest level of clustering?

**DOI:** 10.1186/s12874-016-0127-1

**Published:** 2016-03-08

**Authors:** Christian Bottomley, Matthew J. Kirby, Steve W. Lindsay, Neal Alexander

**Affiliations:** MRC Tropical Epidemiology Group, London School of Hygiene & Tropical Medicine, Keppel Street, London, UK

**Keywords:** Clustering, Multilevel, Hierarchical, Sandwich estimator, Robust variance estimate, Logistic regression

## Abstract

**Background:**

Clustering commonly affects the uncertainty of parameter estimates in epidemiological studies. Cluster-robust variance estimates (CRVE) are used to construct confidence intervals that account for single-level clustering, and are easily implemented in standard software. When data are clustered at more than one level (e.g. village and household) the level for the CRVE must be chosen. CRVE are consistent when used at the higher level of clustering (village), but since there are fewer clusters at the higher level, and consistency is an asymptotic property, there may be circumstances under which coverage is better from lower- rather than higher-level CRVE. Here we assess the relative importance of adjusting for clustering at the higher and lower level in a logistic regression model.

**Methods:**

We performed a simulation study in which the coverage of 95 % confidence intervals was compared between adjustments at the higher and lower levels.

**Results:**

Confidence intervals adjusted for the higher level of clustering had coverage close to 95 %, even when there were few clusters, provided that the intra-cluster correlation of the predictor was less than 0.5 for models with a single predictor and less than 0.2 for models with multiple predictors.

**Conclusions:**

When there are multiple levels of clustering it is generally preferable to use confidence intervals that account for the highest level of clustering. This only fails if there are few clusters at this level and the intra-cluster correlation of the predictor is high.

**Electronic supplementary material:**

The online version of this article (doi:10.1186/s12874-016-0127-1) contains supplementary material, which is available to authorized users.

## Background

Observations are often grouped in assortative clusters, so that two observations from the same cluster tend to be more similar than two selected at random. For example, members of the same household might share genetic and environmental risk factors such that the presence of a disease in one member is predictive of that in others in the same household.

Clustering can influence the amount of uncertainty in parameter estimates. For the sample mean, the standard estimate of the variance must be inflated by a factor 1+*ρ*(*n*−1), where *ρ* is the intra-cluster correlation, which equals the ratio of the variance of cluster means to the total variance of the observations [1], and *n* is the number of clusters. For measures of association between an outcome (*y*) and predictor (*x*) the effect of clustering in the outcome is complicated by the distribution of *x* across clusters—i.e., the degree of clustering in *x*—and it may not always inflate the variance. In a linear regression model the variance of the regression coefficient associated with the predictor is increased by 1+(*n*−1)*ρ*_*x*_*ρ*_*y*_ relative to the OLS estimate [2, 3]. Thus clustering has no effect when either *ρ*_*x*_ or *ρ*_*y*_ is zero and a large effect when both are close to one.

Generally, parameter estimates from generalised linear models, such as logistic regression, are consistent in the presence of clustering, provided that the relationship between the mean of the outcome and the predictor variables is correctly specified. But the standard variance estimates of the regression parameters that ignore clustering are not consistent, and therefore confidence intervals that are based on these variance estimates are incorrect [4]. Fortunately, it is possible to obtain consistent variance estimates for regression parameters using cluster-robust variance estimates (CRVE), which are consistent irrespective of the correlation structure within clusters, provided that observations between clusters are independent [4]. In particular, when there is more than one level of clustering (e.g., individuals clustered in households and households clustered in villages), then CRVE applied at the higher level are consistent, despite the correlation structure within the higher-level clusters (villages) being complicated by correlations within the lower-level clusters (households). Thus a researcher who is faced with multiple levels of clustering can obtain consistent confidence intervals by using CRVE at the highest level of clustering: Angrist and Pischke refer to this as ‘passing the clustering buck’ to the higher level [5].

Consistency, however, guarantees lack of bias only asymptotically, i.e., for sufficiently large sample sizes. Unfortunately, CRVE are biased when there are few clusters. Furthermore, the bias is usually downward so that confidence intervals are too narrow [6]. There is therefore a trade-off. At the lower level of clustering there will be more clusters, but observations from different clusters will be dependent. While, at the higher level, observations from different clusters are more likely to be independent but there will be fewer clusters and the CRVE will be biased.

In this study we explore this trade-off in the context of logistic regression. We use a random effects (conditional) model to simulate binary data that are clustered at two levels, and fit a marginal model to these data, using CRVE to adjust for clustering at either the higher or the lower level. Before we present the simulation, we describe the relationship between marginal and conditional models, and discuss the intra-cluster correlation as a measure of the degree of clustering.

## Methods

### Marginal and conditional models

We model the relationship between a binary outcome and a set of binary predictors in the presence of nested clusters, where the disease and predictors can vary in prevalence between clusters. For example, we might want to predict the probability of a disease based on certain risk factors, and the disease and risk factors are known to cluster in households and villages. In this example, households are the lower-level clusters, and they are nested in villages because members of a household belong to the same village.

One approach used to account for clustering is to include random effects in the regression model. For example, we might model the effects of household and village as independent, normally distributed random variables *z*_*jk*_ and *u*_*k*_ and include these, together with the predictors *x*_1_,…,*x*_*p*_, in the model

(1) $$ {\begin{aligned} \text{log} \left(\frac{\pi_{ijk}}{1-\pi_{ijk}} \right)=\alpha_{0}+\alpha_{1} x_{1ijk}+ \alpha_{2} x_{2ijk}+\ldots+\alpha_{p} x_{pijk}+u_{k}+z_{jk} \end{aligned}}  $$

where *π*_*ijk*_ is the probability of disease in individual *i* from household *j* and village *k*.

We refer to this as the conditional model as the parameter estimates for the predictor variables are conditional on the village and household effects. The model can be fitted by integrating the likelihood over the distribution of the unobserved random effects of village and household, and then maximising this marginal likelihood. A drawback of this approach is that it is necessary to assume distributions for the random effects, and the parameter estimates can be sensitive to the choice of distribution [7].

Alternatively, we can fit a marginal, or population average, logistic regression model that ignores clustering

(2) $$ \text{log} \left(\frac{\pi_{ijk}}{1-\pi_{ijk}} \right)=\beta_{0}+\beta_{1} x_{1ijk} + \beta_{2} x_{2ijk}+\ldots+\beta_{p} x_{pijk}.  $$

The parameters of this model can be estimated by fitting the model using maximum likelihood, ignoring the cluster effects. This is equivalent to solving a set of estimating equations (Eq. A-2 in the Appendix) that have been derived by setting the derivative of the log-likelihood to zero. Each parameter estimates is consistent, provided that the relationship between the probability of disease and predictor variables is correctly specified, but the usual variance estimate based on the second derivative of the log-likelihood is not correct. For a single level of clustering, a cluster robust variance estimate (CRVE) can be used instead (see appendix). This estimate is *unbiased* as the number of clusters tends to infinity, but may be biased when the number of clusters is small. When there are multiple levels of clustering, a consistent variance estimate can be obtained by adjusting for clustering at the higher level—this implicitly accounts for lower-level clustering—but, since the number of higher-level clusters is often small, bias maybe a concern.

The parameters, apart from the intercept, represent log odds ratios in both models. But they are interpreted differently in the two models. For example, for a single, binary predictor, *x*_1_, *β*_1_ is the difference in log odds comparing individuals with *x*_1_=1 and *x*_1_=0 across the whole population; while *α*_1_ is the difference comparing *x*=1 and *x*=0 within a household. The odds ratio, unlike the risk difference and risk ratio, is not collapsible across strata, therefore *α*_1_ and *β*_1_ will be different unless *α*_1_=*β*_1_=0 or there is no variation between households and villages in disease risk.

In general, the relationship between the two sets of parameters can be derived by imagining a dataset that consists of the entire population, and that is generated by the random effects model. The parameters of the marginal model are the ‘estimates’ that are obtained when the marginal model is fitted to this dataset. Mathematically, this is equivalent to solving equation A-2 in the appendix, after replacing *Y*_*ij*_ with *E*_*α*_[*Y*_*ij*_|*x*_*ij*_]=*π*_*ij*_ [8]. Using this approach, Zeger et al. [8] derive the following relationship

(3) $$ \beta \approx \alpha \left(1+c^{2}\left({\sigma_{h}^{2}}+{\sigma_{v}^{2}}\right)\right)^{-1/2}  $$

where *α* is the vector of parameters from the random effects model, *β* is the vector of parameters from the marginal model and $c=16\sqrt {3}/(15\pi)$. From equation 3, it can be seen the odds ratio is closer to the null in the marginal model than the random effects model, and the magnitude of the difference between the odds ratios depends on the amount of variation between clusters, both at the level of the household and the village.

### Intra-cluster correlation

The variance of a regression parameter estimate depends on the amount of clustering in both the outcome and the predictor. The intra-cluster correlation, defined as the correlation between two observations from the same cluster, can be used to quantify the degree of clustering in both variables. Mathematically, it is defined as

(4) $$ \rho=\frac{\mathrm{E}(Z_{ij}-\mu)\left(Z_{i^{*}j}-\mu\right)}{\mathrm{E}\left(Z_{ij} - \mu\right)^{2}} \hspace{1cm} i^{*} \neq i  $$

where *μ* is the overall mean and the expectation is taken over all clusters and pairs of observations within clusters [1]. Assuming that observations are independent conditional on the cluster

(5) $$ \rho=\frac{\mathrm{E}\left(\mu_{j}-\mu\right)^{2}}{\mathrm{E}(Z_{ij}-\mu)^{2}}  $$

where *μ*_*j*_ is the mean for cluster *j*. Therefore *ρ* represents the ratio of the variance in cluster means to the overall variance of the observations.

By definition, *ρ*=1 for cluster-level variables because all the variation is then between clusters, but *ρ* is less than 1 when variables pertain to lower-level units. For example, in a study where data are collected from different villages, village size would be a cluster-level variable with *ρ*=1, but for household and individual-level variables *ρ*<1. In fact, the intra cluster correlation is usually considerably less than 1 for observations made on lower level units. In a survey of binary and continuous outcomes recorded in cluster-based studies conducted in primary care the median intra-cluster correlation was 0.01 and 90 % were less than 0.055 [9].

The intra-cluster correlation of the *outcome* can be calculated directly from the random effects model (Eq. 1) for given values of the parameters and covariate. The intra-cluster correlation can also be calculated for each of the *predictors*, but in this case since these are not defined by a stochastic model it is calculated based on an empirical version of Eq. 4. Note that Eq. 5 implies that *ρ*≥0, but for the predictors the intra-cluster correlation is calculated from the sample rather than the model, consequently the independence assumption necessary for Eq. 5 is not met and the intra-cluster correlation is not necessarily positive. In fact, it reaches a lower bound of −1/(*n*−1) when the prevalence of the predictor is the same in each of *n* clusters [3]. We will use the notation *ρ*_*y*_ to denote intra-cluster correlation defined by the stochastic model for the outcome and $\hat {\rho }_{x}$ to denote the empirical intra-cluster correlation of a predictor.

### Simulation

We conducted a simulation study to explore the coverage of confidence intervals for the parameters of the marginal model. The parameter values used in the simulation are given in Table 1, and we estimated coverage for every combination of these parameters.

**Table 1:** Parameter values

Parameter	Description	Values or range
*α*_0_	Log odds when *x*_*ijk*_=*u*_*k*_=*z*_*jk*_=0	log(0.1/0.9), log(0.2/0.8)
*α*_1_…*α*_*p*_	Conditional log odds ratios	log(1.1), log(2), log(5)
*σ*_*h*_	SD of household effect	log(1.1), log(2), log(5)
*σ*_*v*_	SD of village effect	log(1.05)–log(5)
*I*	No. individuals per household	5, 20
*J*	No. households per village	20, 100
*K*	No. of villages	5, 20

For each parameter combination, we estimated coverage by simulating 10,000 samples from the population using the conditional model (Eq. 1). The marginal model (Eq. 2) was fitted to each sample, and we calculated confidence intervals unadjusted for clustering (CI ^(*un*)^), and intervals adjusted for clustering within households (CI ^(*hh*)^) and villages (CI ^(*vil*)^). We estimated the coverage for each type of interval by calculating the proportion of the 10,000 intervals that contained the true marginal log odds ratio, which was calculated by solving Eq. A-2 in the Appendix with *Y*_*ij*_ replaced by *E*_*α*_[*Y*_*ij*_|*x*_*ij*_]=*π*_*ij*_ (see previous section on marginal and conditional models).

We used predictors of the outcome that varied in their degree of clustering within households and villages. At the extremes, we explored predictors where the proportion positive for the predictor was the same in each village such that $\hat {\rho }_{x}^{(vil)} =-1/(n-1)$, and predictors where the village consists entirely of positives or negatives $\hat {\rho }_{x}^{(vil)}=1$. Table 2 shows, for each predictor, the proportion of individuals positive in each village. We used both household, *x*_1_−*x*_4_, and individual-level, *x*_5_−*x*_7_ predictors. The former are the same for all members of the household (e.g., household income) and the latter vary between household members (e.g., age). We fitted models with a single predictor and also multivariable models that included all the predictors simultaneously.

**Table 2:** Distribution of predictors (*x*_1_- *x*_7_) across villages (V1-V5) and the resulting intra-cluster correlation of the predictor

	Proportion of individuals positive					Intra-cluster correlation $\hat {\rho }_{x}^{(vil)}$
Predictor	V1	V2	V3	V4	V5	
*x*_1_,*x*_5_	0.2	0.2	0.2	0.2	0.2	–0.01
*x*_2_,*x*_6_	0	0.1	0.1	0.3	0.5	0.19
*x*_3_,*x*_7_	0	0.05	0.1	0.1	0.75	0.48
*x*_4_	0	0	0	0	1	1

N.B. In the simulation with 20 villages we created 4 identical sets of villages using the proportions for V1-V5

CI ^(*hh*)^ and CI ^(*vil*)^ were calculated using CRVE (see Appendix) with two corrections to adjust for downward bias. First, the CRVE was inflated by a factor of *n*/(*n*−1), where *n* is the number of clusters. Second, the confidence interval was calculated using a *t*-distribution with *n*−1 degrees of freedom as the reference distribution rather than a standard normal distribution.

We did the simulations in R [10] using the rms package [11] to fit logistic regression models and calculate CRVE.

## Results

### Simulation results

**Fig. 1:**
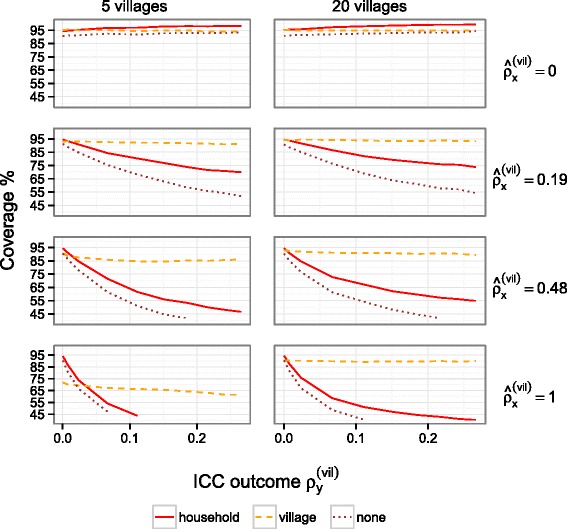
Coverage of 95 % confidence intervals for the log odds ratio of a household-level predictor. The lines correspond to coverage of confidence intervals that adjust for clustering at the village-level, household-level or do not adjust for clustering. Coverage is presented as a function of the degree of village-level clustering in the outcome as measured by the intra-cluster correlation (ICC). The intra-cluster correlation of the predictor ranges between 0 (*top*) and 1 (*bottom*), for *K*=5 (*left*) or *K*=20 (*right*) villages. The remaining parameter values are *α*_0_=*log*(0.1/0.9), *α*_1_=*σ*_*h*_=log(2), *I*=5, *J*=20

CI ^(*un*)^ and CI ^(*hh*)^ had close to 95 *%* coverage when there was limited village-level clustering in the outcome or predictor, but for both coverage decreased as clustering in the outcome and predictor increased (Fig. 1). CI ^(*vil*)^ performed well when the number of villages was large, and also when the number of villages was small (*K*=5), provided that the predictor was not too strongly clustered at the village-level. For example, coverage was more than 85 % for $\hat {\rho }_{x}^{(vil)}<0.5$, in models with a single predictor, and in models that included all predictors simultaneously it was more than 85 % for $\hat {\rho }_{x}^{(vil)}<0.2$. CI ^(*vil*)^ was only outperformed by CI ^(*hh*)^ when there was limited village-level clustering in the outcome $(\rho _{y}^{(vil)}<0.02)$ and the intra-cluster correlation of the predictor was close to 1 $(\hat {\rho }_{x}^{(vil)}\approx 1)$.

Our findings were similar irrespective of whether a household (Fig. 1) or individual-level predictor (Additional file 1: Figure S1) was used. They were also similar when all predictors (individual and household-level) were included in the model simultaneously (Additional file 2: Figure S2 and Additional file 3: Figure S3), although the coverage of CI ^(*vil*)^ was less good.

### Example

We illustrate our findings by analysing data from a randomised trial of a house screening intervention to reduce malaria in children 6 months to 10 years [12]. The intervention was evaluated in terms of its impact on the numbers of mosquitoes caught, anaemia and malaria parasitaemia. The study also collected data on risk factors for malaria, including bed net use. Here we will focus on the presence of malaria parasites in the child, and estimate its association with bed net use and house screening. We use data from the six largest villages (or residential blocks in urban areas) collected on 428 children living in 209 households. The protocol was approved by the Health Services and Public Health Research Board of the MRC UK and The Gambia Government and MRC Laboratories Joint Ethics Committee, and the Ethics Advisory Committee of Durham University. All participants provided consent.

At household-level, malaria was strongly clustered, as were the two predictors: the intracluster correlation was 0.47 for malaria, 0.79 for bed net use and 1 for screening (by design). At the village-level, malaria and bed net use were strongly clustered (intra-cluster correlations 0.28 and 0.33), but screening was not clustered because it was randomly allocated to households.

The odds ratio for screening was 1.13 and the 95 % confidence interval adjusted for household clustering was 0.55 to 2.31. Since there are many households and screening is uncorrelated with village, we expect the coverage of CI ^(*hh*)^ to be close to 95 %.

The odds ratio for bed net use was 0.90. The confidence interval adjusted for household clustering was 0.50 to 1.63 and adjusted for village clustering it was 0.30 to 2.76. Because malaria and bed net use are both highly clustered at the village-level, we expect that CI ^(*vil*)^ will have better coverage than CI ^(*hh*)^.

To further explore the difference between coverage of the two confidence intervals, we fitted a random effects model to the malaria data with bed net use as the predictor. We then simulated samples from this model to estimate the coverage of CI ^(*hh*)^ and CI ^(*vil*)^ for the marginal odds ratio, using the approach described in the previous section. As predicted, we found that the coverage of CI ^(*hh*)^ (68 %) was considerably worse than CI ^(*vil*)^, which had reasonable coverage (85 %), despite the small number of villages.

## Discussion

In general, we recommend using CRVE to adjust for clustering at the higher level. From simulation studies, we found that generally the coverage was better when confidence intervals were adjusted for the higher level of clustering. Adjusting for the lower level of clustering only gave better coverage (i.e., a higher proportion of confidence intervals included the true odds ratio) when the number of higher-level clusters was small *and* the intra cluster correlation of the predictor at this level was close to 1. Neither adjustment produced satisfactory coverage when, at the higher level, there were few clusters and the outcome and predictor were both highly correlated with cluster.

We used two simple adjustments to improve the coverage of confidence intervals: the variance estimate was multiplied by *n*/(*n*−1), and the *t*-distribution with *n*−1 degrees of freedom was used as the reference distribution rather than the standard normal distribution. Both adjustments are implemented in the svyset command in Stata. Other methods for adjusting confidence intervals might give better coverage, but are not currently implemented in routine software. Pan and Wall [13] suggest modifying the degrees of freedom used for the reference *t*-distribution, and a number of authors have proposed methods for correcting for the bias in the variance estimates [14–16]. Bootstrap methods, in which clusters are resampled with replacement, offer another approach, but do not perform better than CRVE [17].

The results we have presented here are from simulation, rather than algebraic demonstration. Nevertheless the simulations cover wide ranges of the key parameters— *ρ*_*x*_ and *ρ*_*y*_ at the higher level of clustering—and our conclusions were not sensitive to the values used for the other parameters, except the number of higher level clusters. For this parameter we present results for a small (*K*=5) and large (*K*=20) number. At *K*=20 the coverage was close to 95 % when confidence intervals were adjusted for higher-level clustering, and we expect coverage to improve if *K*>20. We chose not to explore with further granularity the region of parameter space where adjustment at the lower level of clustering is favourable (high *ρ*_*x*_, low *ρ*_*y*_ at the higher level of clustering and a small number of clusters at this level) because the region is small and the lower level has only a slight advantage here.

We have explored the performance of standard errors adjusted for clustering without adjusting the log odds ratio. In the framework of Generalised Estimating Equations (GEE) this is equivalent to assuming an ‘independence’ working correlation matrix. The log odds ratio can be estimated more efficiently (i.e., with smaller asymptotic variance) if the correlation structure is used to inform the estimate. For a single level of clustering, a constant correlation between observations from the same cluster is often assumed—the so-called ‘exchangeable’ correlation structure. When there are multiple levels of clustering one could assume a constant correlation at the higher-level, but this is a crude approximation because the correlation between observations from the same higher-level cluster will depend on whether they also come from the same lower-level cluster. Several authors have therefore modelled the correlation structure that occurs when there is multi-level clustering and have demonstrated that this gives more efficient estimates compared to either the independence or the exchangeable structure [18–20]. While these methods provide benefit in terms of efficiency, the complicated correlation structure is not easily implemented in standard software, and the additional parameters can lead to problems with convergence, particularly when the number of cluster is small [20]. Furthermore, the loss of efficiency that results from assuming an independence structure is generally small [4, 21], except when the intra-cluster correlation of the outcome is large (*ρ*_*y*_>0.3) and the predictor varies within clusters [22]. The relative simplicity of assuming an ‘independence’ correlation structure (i.e., the CRVE approach discussed in this manuscript) might therefore remain attractive to the applied researcher, even if the resulting estimate it is not the most efficient.

## Conclusions

CRVE are commonly used to construct confidence intervals that take account of clustering. When clustering occurs at multiple levels, CRVE can be used at the higher level of clustering, except if there are few clusters at this level and the intra-cluster correlation of the predictor is high.

## Appendix

In a logistic regression model, the relationship between a binary variable *Y*_*ij*_ and predictors *x*_1*ij*_,…,*x*_*pij*_ is

(A-1) $$\begin{array}{@{}rcl@{}} \text{log} \left(\frac{\pi_{ij}}{1-\pi_{ij}} \right)&=&\beta_{0} + \beta_{1}x_{1ij}+\beta_{2} x_{2ij} +\ldots+\beta_{p} x_{pij}  \\ &=& x'_{ij} \beta \end{array} $$

where *π*_*ij*_=P (*Y*_*ij*_=1|*x*_*ij*_) for observation *i* from cluster *j*.

Assuming responses are independent, the maximum likelihood estimate, $\hat {\beta }$, is the solution to the equations

(A-2) $$ U(\beta)=\sum_{j=1}^{n} X_{j}'\left(Y_{j}-\pi_{j}(\beta)\right)=0  $$

where *Y*_*j*_ is a column vector of responses in cluster *j*, and *X*_*j*_ is matrix whose columns are the predictors of *Y*_*j*_. Since the *Y*_*j*_ are independent, it can be shown by the central limit theorem and using a Taylor expansion that, asymptotically, as the number of clusters (*n*) tends to infinity, $\hat {\beta }$ is normally distributed with mean *β* and variance

$$ \left(\frac{\partial U'}{\partial \beta} \right)^{-1} \text{Var}(U(\beta)) \left(\frac{\partial U'}{\partial \beta} \right)^{-1} $$

where $\text {Var}(U(\beta))=\sum _{j} X_{j}' \text {Var}(Y_{j}) X_{j} $, $\frac {\partial U'}{\partial \beta }=\sum _{j} X_{j}' V_{j} X_{j}$ and *V*_*j*_ is a diagonal matrix with elements *π*_*ij*_(1−*π*_*ij*_).

The so-called sandwich estimator, which is also referred to as the cluster robust variance estimate (CRVE), is obtained by replacing *π*_*ij*_ in *V*_*j*_ with $\hat {\pi }_{ij}$ and using $(y_{j}-\hat {\pi }_{j})(y_{j} -\hat {\pi }_{j})'$ to estimate the covariance matrix Var(*Y*_*j*_).

## Additional files

Additional file 1
**Figure S1.** Coverage of 95 % confidence intervals for the log odds ratio of an individual-level predictor. The intra-cluster correlation of the predictor ranges between 0 (top) and 1 (bottom), for *K*=5 (left) or *K*=20 (right) villages. The remaining parameter values are *α*_0_=*log*(0.1/0.9), *α*_1_=*σ*_*h*_=log(2), *I*=5, *J*=20. (TIFF 30617 kb)

Additional file 2
**Figure S2.** Coverage of 95 % confidence intervals for the log odds ratio of a household-level predictor from a logistic regression model that includes multiple predictors (*x*1−*x*7). The intra-cluster correlation of the predictor ranges between 0 (top) and 1 (bottom), for *K*=5 (left) or *K*=20 (right) villages. The remaining parameter values are *α*_0_=*log*(0.1/0.9), *α*_1_=⋯=*α*_7_=*σ*_*h*_=log(2), *I*=5, *J*=20. (TIFF 30617 kb)

Additional file 3
**Figure S3.** Coverage of 95 % confidence intervals for the log odds ratio of an individual-level predictor from a logistic regression model that includes multiple predictors (*x*1−*x*7). The intra-cluster correlation of the predictor ranges between 0 (top) and 1 (bottom), for *K*=5 (left) or *K*=20 (right) villages. The remaining parameter values are *α*_0_=*log*(0.1/0.9), *α*_1_=⋯=*α*_7_=*σ*_*h*_=log(2), *I*=5, *J*=20. (TIFF 30617 kb)

## Abbreviations

CRVE: Cluster robust variance estimate
OLS: Ordinary least squares
CI: Confidence interval
GEE: Generalised estimating equation
SD: Standard deviation
