# Supplementary figures and images for: Can the buck always be passed to the highest level of clustering?
Source: BMC Med Res Methodol. 2016 Mar 8;16:29. doi: 10.1186/s12874-016-0127-1 (PMC4784323; doi:10.1186/s12874-016-0127-1)

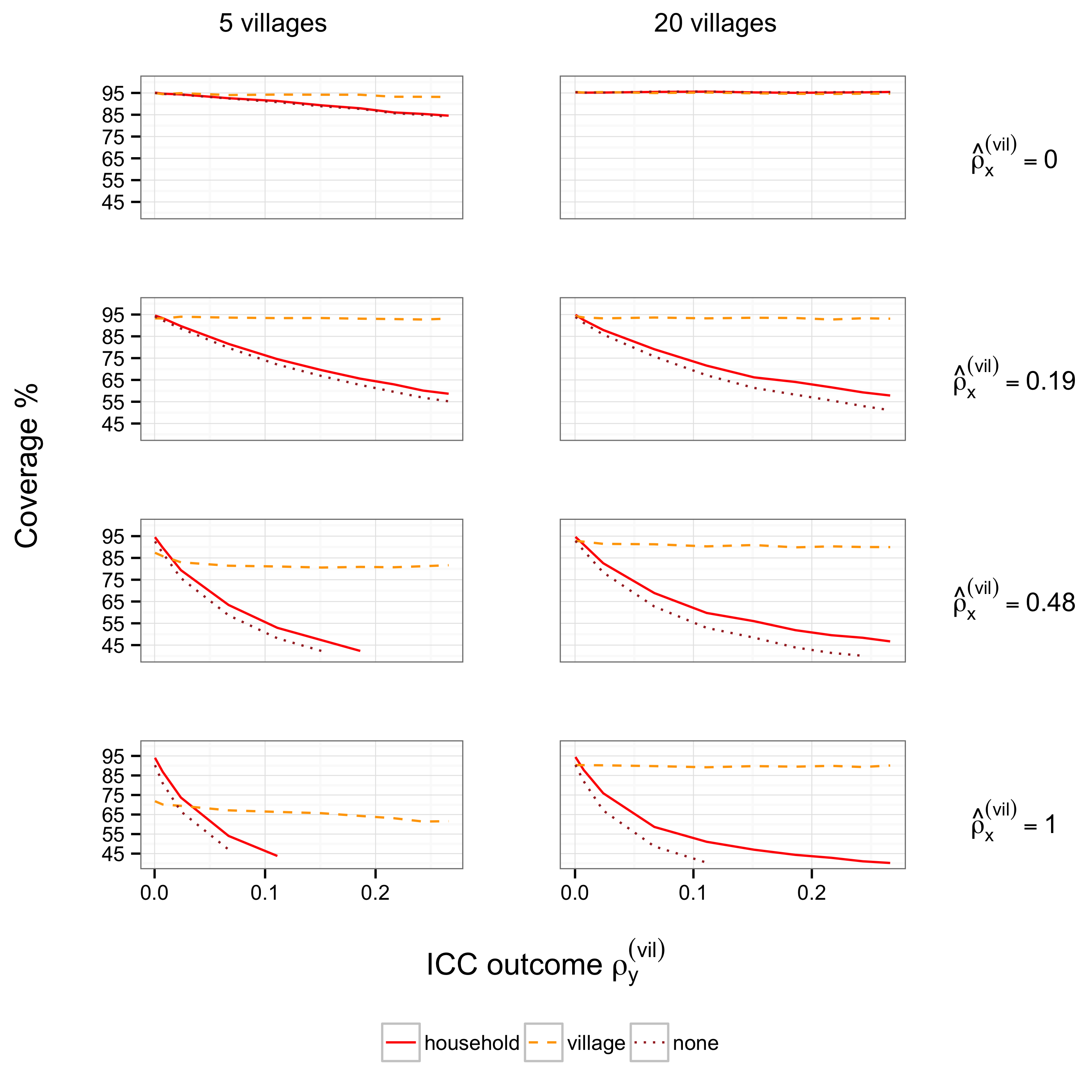

Supplement: Additional file 1 — Figure S1. Coverage of 95 % confidence intervals for the log odds ratio of an individual-level predictor. The intra-cluster correlation of the predictor ranges between 0 (top) and 1 (bottom), for K=5 (left) or K=20 (right) villages. The remaining parameter values are α 0=log(0.1/0.9), α 1=σ h=log(2), I=5, J=20. (TIFF 30617 kb) [file 12874_2016_127_MOESM1_ESM.tiff]

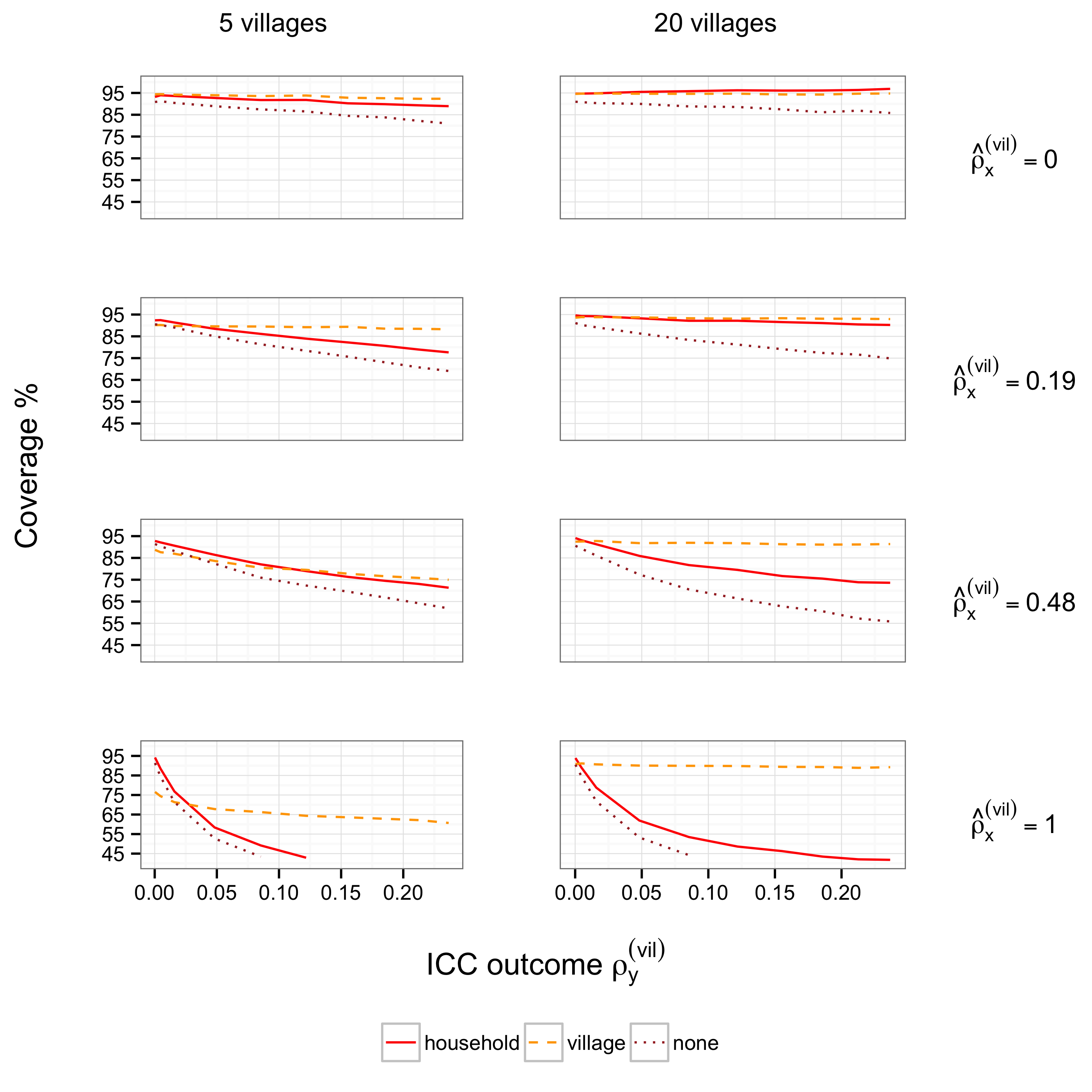

Supplement: Additional file 2 — Figure S2. Coverage of 95 % confidence intervals for the log odds ratio of a household-level predictor from a logistic regression model that includes multiple predictors (x1−x7). The intra-cluster correlation of the predictor ranges between 0 (top) and 1 (bottom), for K=5 (left) or K=20 (right) villages. The remaining parameter values are α 0=log(0.1/0.9), α 1=⋯=α 7=σ h=log(2), I=5, J=20. (TIFF 30617 kb) [file 12874_2016_127_MOESM2_ESM.tiff]

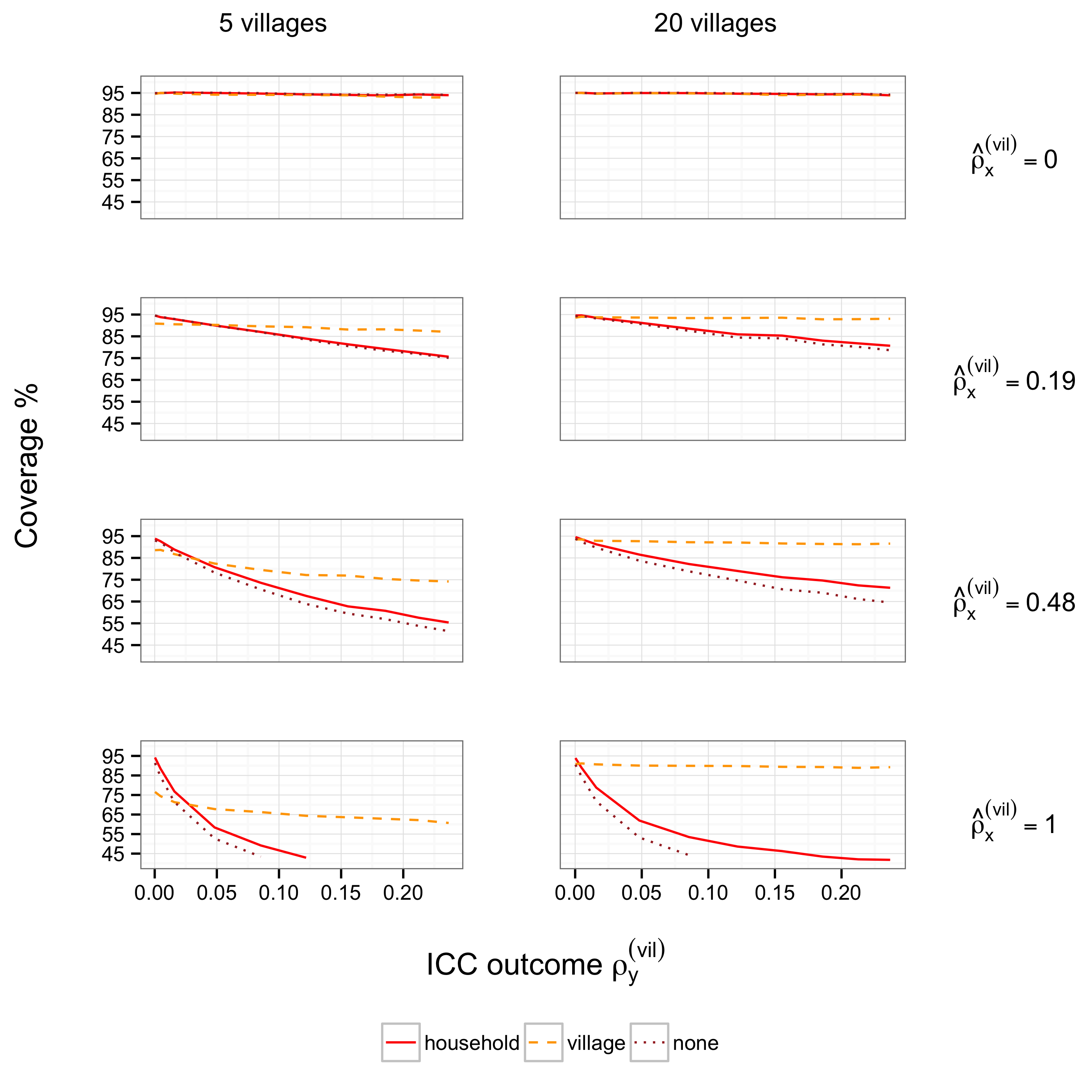

Supplement: Additional file 3 — Figure S3. Coverage of 95 % confidence intervals for the log odds ratio of an individual-level predictor from a logistic regression model that includes multiple predictors (x1−x7). The intra-cluster correlation of the predictor ranges between 0 (top) and 1 (bottom), for K=5 (left) or K=20 (right) villages. The remaining parameter values are α 0=log(0.1/0.9), α 1=⋯=α 7=σ h=log(2), I=5, J=20. (TIFF 30617 kb) [file 12874_2016_127_MOESM3_ESM.tiff]
